# Effect of long-acting β2-agonist and corticosteroid combination treatment on parotid glands of rats (histological and immunohistochemical study)

**DOI:** 10.1186/s12903-025-07344-w

**Published:** 2025-12-05

**Authors:** Lobna Mohamed Nabil, Dalia Riad

**Affiliations:** 1 Lecturer of Oral Biology Department, Faculty of Oral and Dental medicine, Modern University for Technology and Information, Cairo, Egypt; 2 Associate Professor of Biology Department, Faculty of Dentistry, Beni-Suef University, Beni Suef, Egypt

**Keywords:** COPD, Long-acting β2-agonist, Corticosteroid, α-SMA, Parotid glands

## Abstract

**Background:**

Bronchial asthma is a common chronic condition frequently managed with corticosteroids, β2 receptor agonists, or their combination. While these therapies effectively control respiratory symptoms, emerging evidence suggests they may adversely affect oral and salivary gland health. However, most existing studies focus on inhalation-related tooth erosion or xerostomia, with limited histological evidence on glandular changes. This study uniquely investigates the direct impact of combination therapy of salmeterol and fluticasone propionate on the parotid glands using histological and immunohistochemical analyses.

**Objective:**

to evaluate the possible effect of long-acting β2-agonist (Salmeterol) and corticosteroid (Fluticasone propionate) combination treatment on parotid glands and assess the histological and immunohistochemical changes.

**Materials and methods:**

36 male albino rats were allocated into control and experimental groups. The experimental group received daily intraperitoneal injections of a combination of salmeterol and fluticasone propionate powder at a dosage of 0.55 mg/kg/day, while the control group was administered an equal volume of saline. Nine rats from each group were sacrificed at 14 and 25 days of treatment. The parotid glands were dissected at each time point and subjected to histological examination using H&E and immunohistochemical staining for α-SMA.

**Results:**

Histological examination of group II after 14 days, serous acini appeared spherical with hyperchromatic nuclei, while ducts showed epithelial thinning and stagnant secretion. By 25 days, acini became irregular with cytoplasmic vacuolations, increased spacing, and inflammatory infiltration. Engorged blood vessels were observed. Immunohistochemical results using anti-α-SMA staining proved a significant decrease in myoepithelial activity compared to the control group.

**Conclusions:**

Prolonged combination treatment leads to quantitative and qualitative changes in parotid salivary glands, potentially impacting oral and dental health. Therefore, greater attention should be given to the oral health of individuals using this medication.

## Introduction

 Chronic obstructive pulmonary disease (COPD) is a chronic respiratory condition characterized by chronic airflow obstruction due to structural alterations in the lung, including destruction of lung parenchyma, mucus accumulation within the airways, and inflammation and hyperplasia of the airway epithelium [1, 2]. COPD is commonly associated with an aberrant inflammatory response to inhaled noxious particles and gases, resulting in significant morbidity and mortality [3].

Recent data indicate a significant rise in the incidence of COPD, primarily attributed to air pollution and smoking [4]. The symptoms of COPD contribute to a considerable global burden of respiratory diseases [5]. This condition leads to impaired physical function, an elevated risk of hospitalization, and exacerbation of symptoms such as chronic bronchitis and emphysema, all of which severely compromise the quality of life for affected individuals [6].

Inhaled corticosteroids (ICS) are recommended as first-line therapy for managing inflammation in COPD, while the addition of a long-acting β2 agonist to ICS therapy is advised for patients with inadequate control on ICS alone, providing more effective treatment outcomes [7]. Furthermore, incorporating inhaled long-acting β2 agonists has demonstrated superior efficacy in improving lung function and controlling symptoms and exacerbations, compared to simply increasing the ICS dose in patients with varying degrees of symptom severity. Consequently, this combination therapy has become a widely recognized and recommended treatment approach in international asthma guidelines [8]. According to the Global Initiative for Asthma (GINA), it recommended daily ICS and a long-acting β agonist as combination therapy as a maintenance and reliever therapy for adolescents/adults of varying degrees of asthma severity [9]. The combination of Salmeterol and Fluticasone exerts significant effects in patients with COPD, improving lung function, elevating CD4 + counts and the CD4+/CD8 + ratio, reducing CD8 + levels, and producing minimal changes in the oropharyngeal microflora [10].

Salmeterol (Sal) is a prominent member of the long-acting β2 adrenergic receptor agonist subgroup of bronchodilators, commonly employed in managing COPD. These medications promote the relaxation of airway smooth muscle cells, resulting in bronchodilation [11], and can potentially mitigate early and late-phase antigen-induced airway hyperreactivity [12–14]. Sal is commonly prescribed in combination with glucocorticoids like fluticasone for the treatment of COPD. Sal works by inducing bronchodilation and attenuating airway inflammation through the suppression of inflammatory mediators such as interleukin-4 (IL-4) [15]. Clinical studies have indicated that combining salmeterol and fluticasone propionate enhances lung function and provides better symptom control than equivalent or higher doses of fluticasone propionate alone in COPD patients [16–19].

Patients on long-term medication therapy are prevalent, and it is crucial for dentists to recognize the potential side effects of different drugs and understand the underlying mechanisms by which these medications function. Numerous medications prescribed for the management of chronic respiratory conditions can have adverse effects on oral health. For example, many of these drugs are linked to xerostomia, dental caries, and other oral mucosal lesions. Among the most commonly reported issues associated with medication use is salivary dysfunction, particularly the sensation of dry mouth [20, 21]. While previous research has explored oral complications such as dental caries and mucosal dryness in asthmatic patients, no studies have directly examined salivary gland tissue responses to long-acting β2-agonist and corticosteroid combination therapy. To our knowledge, no study has systematically assessed histopathological and immunohistochemical changes in parotid glands following systemic exposure to this drug combination in an animal model. Our study aims to fill this critical gap.

So, the study aimed to evaluate the possible effect of long-acting β2-agonist (Salmeterol) and corticosteroid (Fluticasone propionate) combination treatment on an albino rat’s parotid glands and assess the histological and immunohistochemical changes.

## Materials and methods

All experimental procedures were carried out according to protocols approved by the Ethics Committee of Research of the Faculty of Dentistry, Ain Shams University, with approval number FDASU-Rec IR 052334.

This study comprised 36 pathogen-free male Albino rats, obtained from the animal house of the Medical and Scientific Research Institute (MASRI) at Ain Shams University. Rats weighing between 200 and 250 g, with an average age of 7 weeks, were maintained under controlled environmental conditions (temperature: 22 ± 2 °C, humidity: 55 ± 10%, and a 12-h light/dark cycle). The animals were housed in wire mesh cages, with five rats per cage. The study adhered to the Five Freedoms of Animal Welfare, which include freedom from hunger or thirst, freedom from discomfort, freedom from pain, freedom to engage in normal behaviors, and freedom from fear and distress. The rats were provided with a balanced diet consisting of fresh vegetables, dried bread, and ad libitum access to tap water. Upon completion of the experiment, the rats were sacrificed by overdose of an intraperitoneal injection of sodium thiopental (80 mg/kg), and the remains were properly disposed of by incineration at Ain Shams Hospital.

- Study Setting: The study was conducted in the animal house of the MASRI at Ain Shams University under the supervision of a specialized veterinarian.
- Sample Size Calculation: The sample size was calculated using the G*Power statistical power analysis program (version 3.1.9.7) [22]. Based on the study of Metwally et al. (2013) [23] and using α = 0.05 and power = 0.8, the total sample size was 36 rats (18 per group).

### Eligibility criteria

#### Inclusion criteria

 Pathogen-free male Albino rats, with an average weight ranging from 200 to 250 g and an average age of 7 weeks, were included in the study.

#### Exclusion criteria

 Rats infected with pathogens, female rats, or those with changes in age or weight were excluded from the study.

- Study Procedure
  Rats were randomly assigned to two groups:

1. Group I (Control Group) (*n* = 18): rats received normal saline.
2. Group II (Treated/Experimental Group) (*n* = 18): rats were administered a combination of long-acting β2-agonist (salmeterol) and corticosteroid (fluticasone propionate) at a ratio of 50 µg: 250 µg, equivalent to 0.55 mg/kg/day, dissolved in normal saline [24].

### Drug administration

Saline was administered to the control group, and Salmeterol, a fluticasone propionate combination, was administered via intraperitoneal injection in the experimental group. The treatment period lasted 25 days [25]. Diclofenac, administered at 5 mg/kg once daily, controlled the pain [26].

### Tissue samples

Nine rats from both the control and experimental groups were randomly selected and sacrificed at 14 and 25 days [23]. The rats were euthanized with an overdose of thiopental sodium (80 mg/kg) via intraperitoneal injection [27]. Randomization and blinding were ensured by assigning coded identifiers to animals before sacrifice. Parotid glands were carefully dissected, rinsed in saline, and fixed in 10% neutral buffered formalin for histological and immunohistochemical analyses (Table 1).

**Table 1:** Showing the experimental design, treatment schedule, and assessment time

Day	Procedure	Control Group (*n* = 18)	Experimental Group (*n* = 18)
0	Random allocation, baseline	Normal saline (i.p.)	Salmeterol + Fluticasone (0.55 mg/kg/day, i.p.)
1–13	Daily treatment	Normal saline	Combination drug
14	Sacrifice (first subgroup, *n* = 9/group)	Histological & α-SMA assessment	Histological & α-SMA assessment
15–24	Continued daily treatment	Normal saline	Combination drug
25	Sacrifice (second subgroup, *n* = 9/group)	Final histological & α-SMA assessment	Final histological & α-SMA assessment

All 36 animals survived the experimental period without loss or exclusion. Therefore, statistical analyses were conducted on the full dataset without the need for imputation or adjustment for missing values.

a  Routine hematoxylin and Eosin staining
  The samples were dehydrated in graded alcohol, cleaned in xylene, and embedded in paraffin. Hematoxylin and eosin staining were performed on Sects. 4–5 thick in preparation for standard histological analysis [28].
b Alfa smooth muscle actin (α-SMA) immune-histochemical staining
  A drop of hydrogen peroxide was applied to the specimens, covering them for 5 min. The specimens were rinsed twice in PBS (pH 7.4) for 5 min each. The sections were then placed in a citrate buffer for 2:30 min, followed by heating in a microwave, surrounded by water jars to prevent boiling, for another 2:30 min. The specimens were cooled and then washed in PBS solution for 3 min. Subsequently, a drop of Super Block was applied to the sections, which were incubated for 5–10 min and rinsed four times in PBS. The sections were then immunolabeled with αSMA using a dilution of 1:3 (Santa Cruz Biotechnology, Inc., Europe), incubated overnight at 37 °C, followed by rinsing in PBS. Afterward, a drop of anti-polyvalent was applied to the sections for 30 min, followed by a PBS rinse. Next, a drop of horseradish peroxidase enzyme was applied to the sections for 30 min, after which they were rinsed four times in PBS for 3 min each. The sections were then covered with a substrate chromagen solution consisting of 4 drops of 3,3’-diaminobenzidine tetrachloride (DAB) chromagen mixed with 5 cm of DAB substrate and rinsed with PBS. The specimens were counterstained with hematoxylin for 30 s until a blue color appeared, then rinsed with distilled water. Subsequently, they were dehydrated in graded alcohol and cleared in xylene. Finally, the specimens were mounted in dibutyl phthalate polystyrene xylene [29].
  Histological and immunohistochemical analyses were performed by two independent examiners blinded to group allocation. Slides were coded and randomized by a technician to ensure unbiased evaluation.

### Image analysis for immunostaining interpretation

Immunostained sections were examined using a Leica Qwin 500 image analysis system (Leica Microsystems, Wetzlar, Germany). α-SMA expression was evaluated both quantitatively (percentage of positive area using ImageJ) and qualitatively (staining intensity categorized as mild, moderate, or strong by blinded observers). Positive immunolabeling was identified as a brown cytoplasmic reaction within myoepithelial cells, assessed at 400× magnification in five random fields per sample.

### Statistical analysis

The statistical analysis of the data obtained from computer image analysis was conducted to assess the percentage area of α-SMA immune expression. Data was checked for normality using the Kolmogorov-Smirnov and Shapiro-Wilk tests. The mean area fraction of α-SMA between the control and experimental groups was compared using one-way analysis of variance (ANOVA). Data were coded and entered using SPSS version 28 (IBM Corp., Armonk, NY, USA). Post hoc pairwise comparisons were performed using Tukey’s test following ANOVA. A p-value of less than 0.05 was considered to indicate statistical significance [30].

## Results

### Histological examination results

**Fig. 1:**
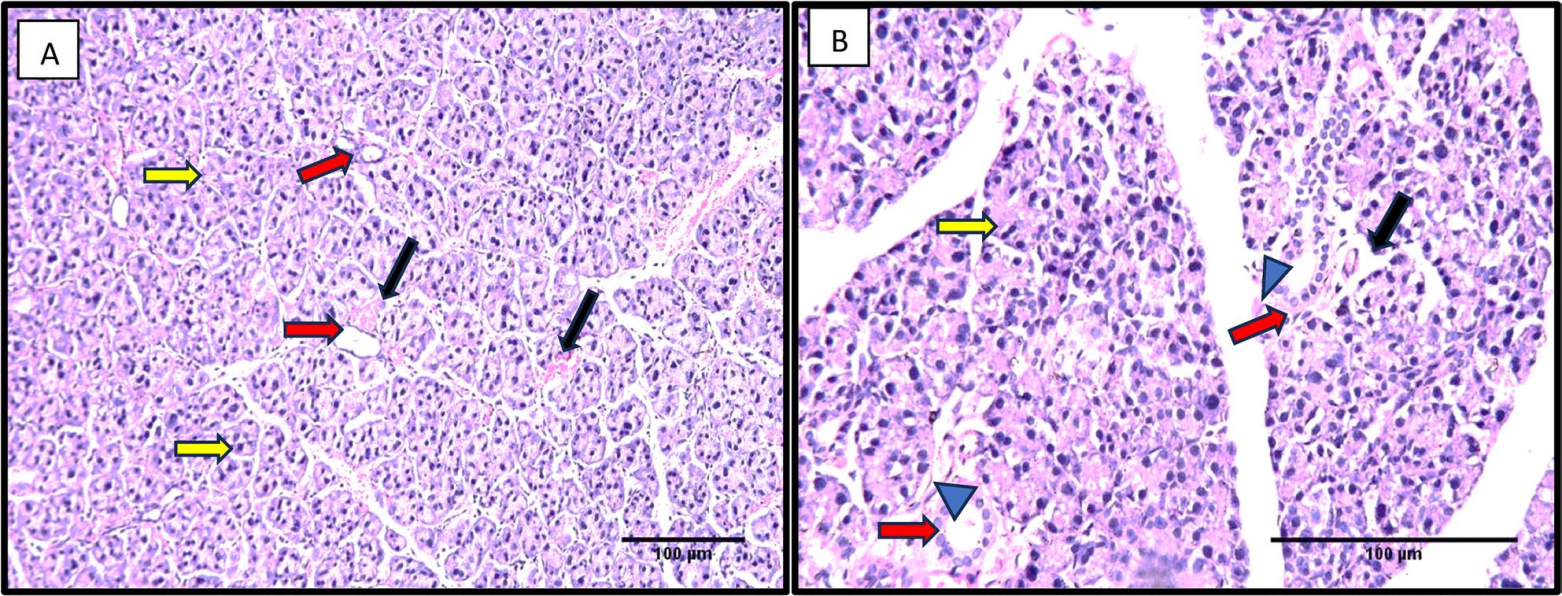
A photomicrograph from Group I showing serous acini that are nearly spherical in shape, exhibiting basally located nuclei (yellow arrows). The striated ducts display a uniform epithelial lining thickness with centrally positioned nuclei (red arrows). The ductal lumens appear wide, and no stagnant secretion (blue arrowheads). Blood vessels show minimal extravasation (black arrow). A B H&E Orig. Mag. **A** 200, **B** 400), Scale bar=100 μm

Examination of histological sections of group I revealed serous acini with a nearly spherical shape and basally situated nuclei. Striated duct lining showed short columnar cells with centrally placed nuclei, regular lining thickness, and a clear lumen (Fig. 1).

**Fig. 2:**
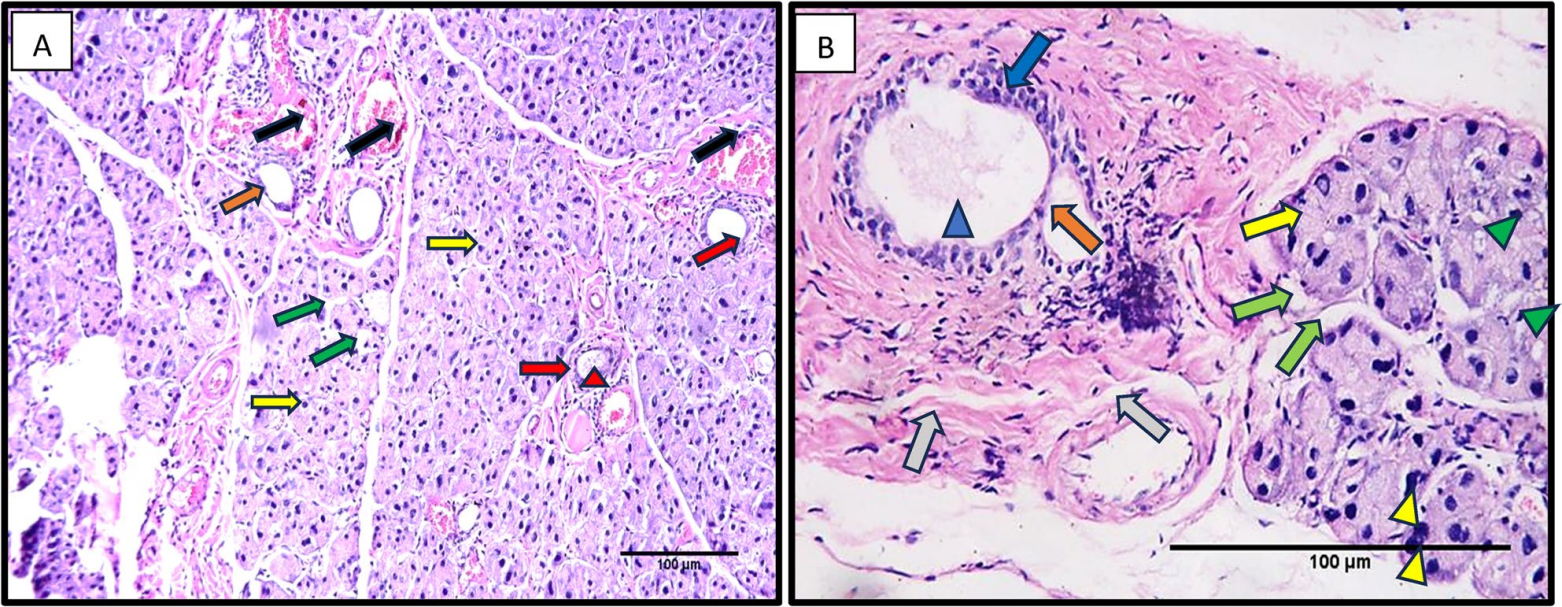
A photomicrograph from Group II (after 14 days) showing serous acini that are predominantly spherical in shape, with basally located nuclei (yellow arrows). Some acini exhibit spacing (green arrows) and hyperchromatic nuclei (yellow arrowheads), while others display mild cytoplasmic vacuolations (green arrowheads). The striated ducts reveal an apparent thinning of the lining epithelium (red arrows) accompanied by mild stagnant luminal secretion (red arrowhead). The excretory ducts show areas of regular pseudostratified epithelium (blue arrow), alternating with regions of epithelial thinning (orange arrows) and mild intraluminal secretion stagnation (blue arrowhead). Blood vessels exhibit evident extravasation (black arrows), and the surrounding fibrous connective tissue appears disorganized (grey arrows).H&E Orig. Mag. **A** 200, **B** 400), Scale bar=100 μm

Serous acini in group II after 14 days appeared nearly spherical in shape with basally situated nuclei. Some acini showed spacing with hyperchromatic nuclei. Striated ducts showed an apparent thinning of the duct lining and mild stagnant secretion. In addition, excretory ducts showed nearly regular pseudostratification at some parts, while an evident epithelium thinning at other parts was observed. Blood vessels showing extravasation were noted (Fig. 2A).

The excretory duct showed an apparent thinning of pseudostratification and mild stagnant secretion. Some acini with spacing in between, hyperchromatic nuclei, and nearly mild cytoplasmic vacuolations were seen, as well as disorganized fibrous connective tissue surrounding the ducts (Fig. 2B).

**Fig. 3:**
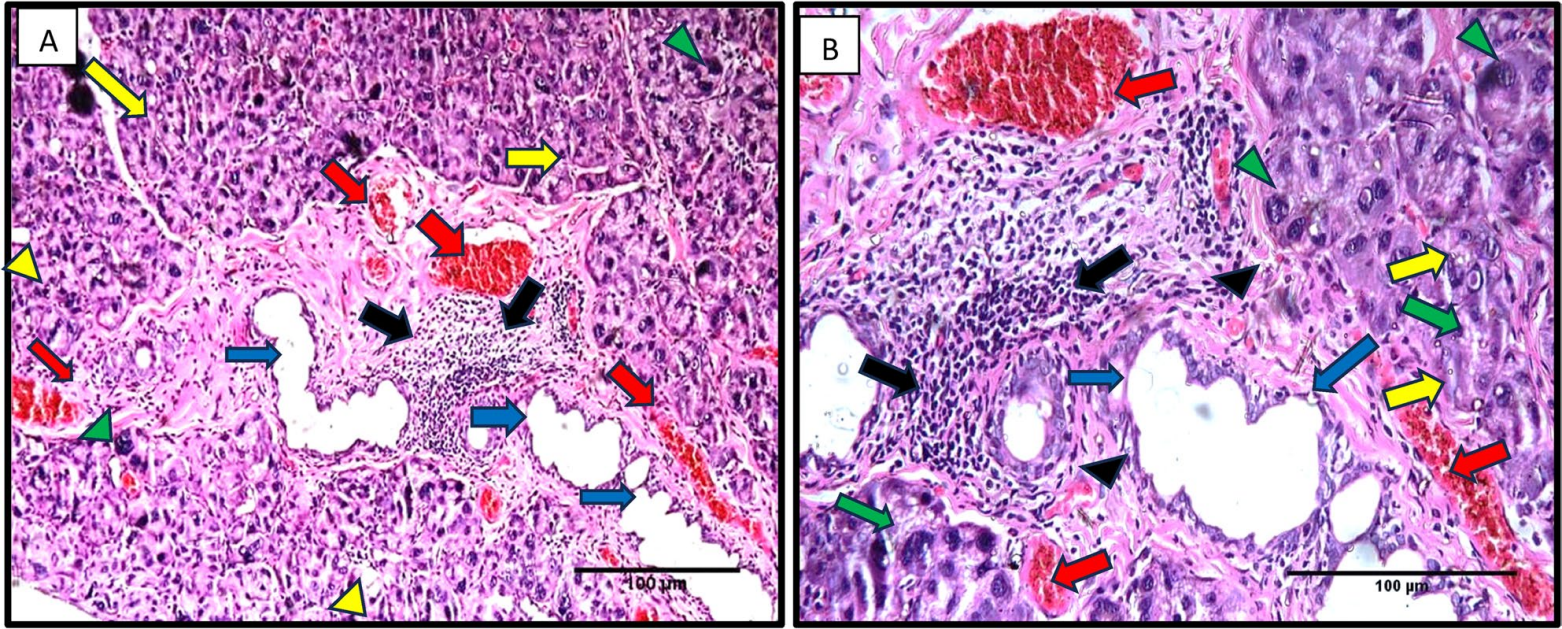
A photomicrograph from Group II (after 25 days) showing serous acini with irregular outlines (yellow arrows), hyperchromatic nuclei (green arrowheads), and noticeable interacinar spacing (yellow arrowheads), along with apparent cytoplasmic vacuolations (green arrows). The excretory ducts exhibit a marked thinning of the epithelial lining (blue arrows). Inflammatory cell infiltration is clearly observed (black arrows), accompanied by multiple engorged blood vessels (red arrows). The fibrous connective tissue surrounding the ducts appears relatively disorganized (black arrowheads). (H&E, original magnification x200). H&E Orig. Mag. **A** 200, **B** 400), Scale bar=100 μm

In group II, after 25 days, serous acini with relatively irregular outlines and hyperchromatic nuclei were observed. Some spacing between serous acini was seen. Excretory ducts showed an apparent thinning of the duct lining from columnar to flat cells. An obvious inflammatory cell infiltration and multiple extravasated blood vessels were observed. Relatively less organized fibrous CT surrounding the ducts was noted. Some serous acini with hyperchromatic nuclei and apparent cytoplasmic vacuolations were seen. Excretory ducts showing an apparent thinning of the duct lining were observed. Apparent inflammatory cell infiltration and engorged blood vessels (Fig. 3).

**Fig. 4:**
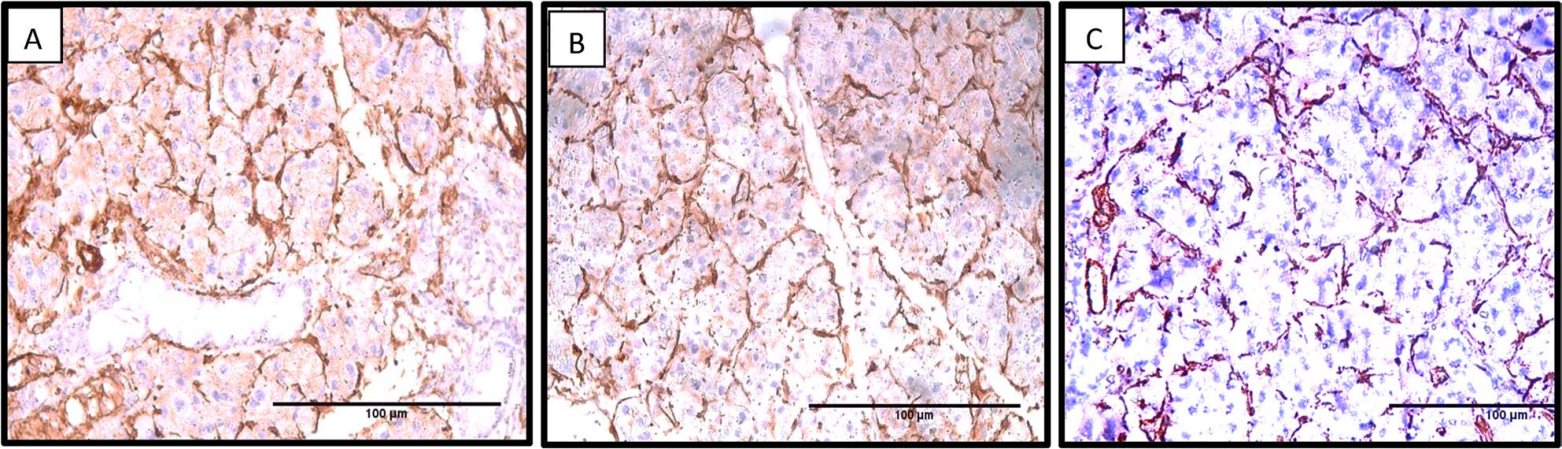
αSM Immunostained sections showing the reactive changes in the parotid gland tissues of all research groups. **A** Group a positive immunoreaction with a strong intensity of myoepithelial cells at the periphery of serous acini, with a similarly intense positive reaction observed in the intercalated ducts. **B** After 14 days, Group II showed moderate intensity of positive immunoreaction of myoepithelial cells at the periphery of serous acini. **C** Group II, after 25 days, showed a mild intensity of myoepithelial cells' positive immunoreaction at the serous acini's periphery. (anti-αSMA, Orig. Mag. 400, Scale bar=100μm)

- Immunohistochemical results
  The αSMA marker was employed to identify active myoepithelial cells. A positive reaction was characterized by brown nuclear and cytoplasmic staining located at the periphery of the serous acini and intercalated ducts.
  In group I, a positive immunoreaction with strong intensity was observed in myoepithelial cells at the periphery of serous acini and the intercalated duct (Fig. 4A).
  Meanwhile, after 14 days, Group II showed a moderate intensity of positive immunoreaction of myoepithelial cells (Fig. 4B).

After 25 days, Group II showed a mild intensity of myoepithelial cell immunoreaction (Fig. 4 C). All histological and immunohistochemical results were summarized in Table 2.

**Table 2:** Showing histological and immunohistochemical results of control and experimental groups

**Group**	** Time Point**	**Histological Results (H&E)**	** Immunohistochemical Results (α-SMA)**
Control (Group I)	14, 25 days	Normal serous acini with spherical shape and basally placed nuclei. Striated ducts with short columnar lining, clear lumen, and normal C.T	Strong α-SMA positivity immunoreactivity of myoepithelial cells at acinar periphery and intercalated ducts.
Experimental (Group II)	14 days	Serous acini are mostly spherical with some spacing and hyperchromatic nuclei. Thinning of striated duct lining, mild stagnant secretion, mild cytoplasmic vacuolations	Moderate α-SMA positivity
Experimental (Group II)	25 days	Irregular acinar outlines, hyperchromatic nuclei, cytoplasmic vacuolation, inter-acinar spacing, epithelial thinning of excretory ducts, inflammatory infiltration, and multiple extravasated vessels.	Mild α-SMA positivity

### Statistical results

**Fig. 5:**
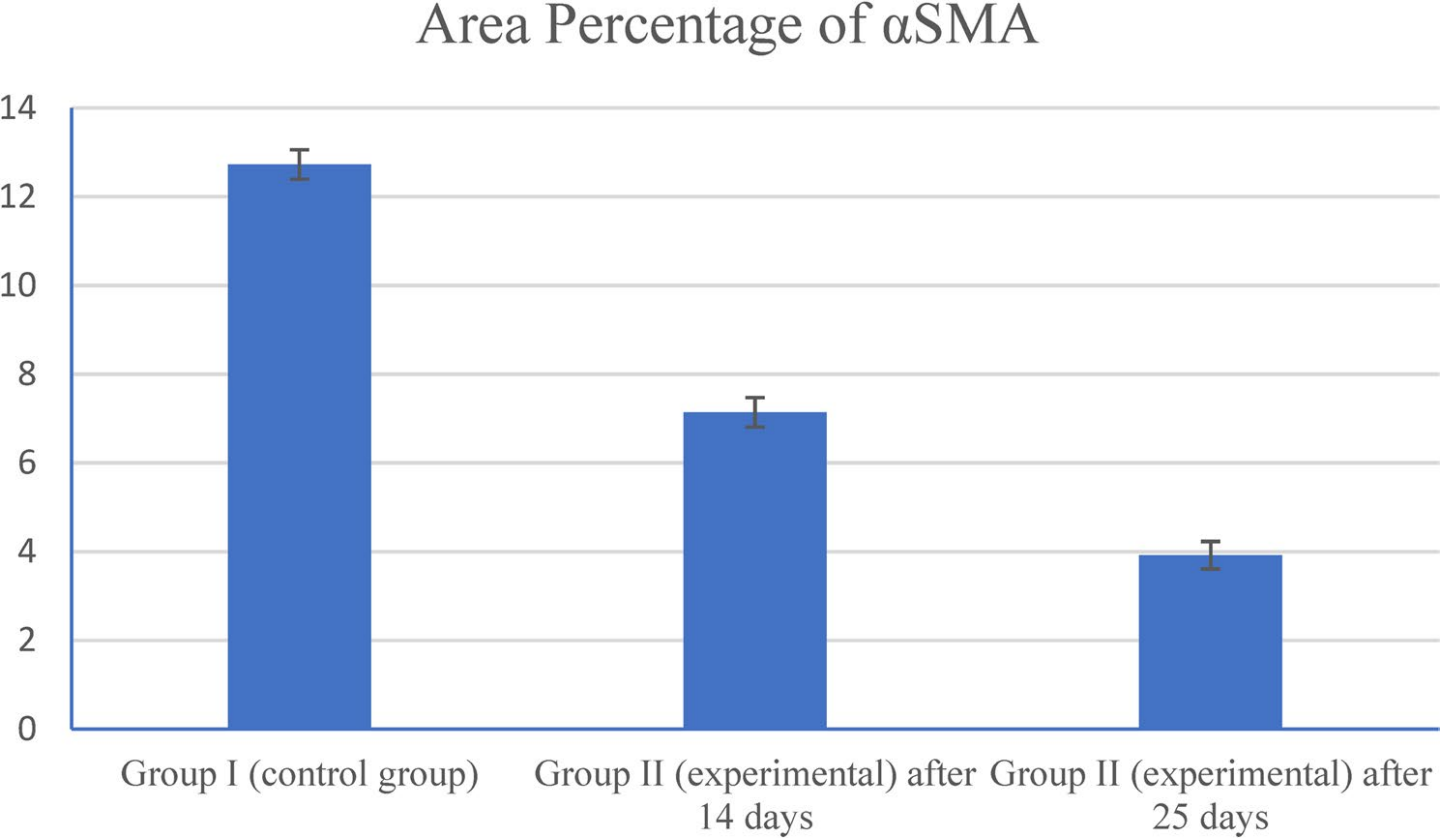
Represents the difference in the mean area percentage of αSMA between all groups

Quantitative analysis revealed that Group I (control) exhibited the highest mean area percentage of αSMA immunopositivity, showing a statistically significant difference when compared with all other groups (*P* < 0.001). In Group II (experimental), αSMA immunopositivity showed a significant decrease after 14 days relative to the control group (*P* < 0.001). A further significant reduction was observed after 25 days compared with the 14-day subgroup (*P* < 0.001). Moreover, the 25-day experimental subgroup demonstrated a highly significant decrease in the mean area percentage of αSMA immunopositivity compared with the control group (*P* < 0.001) (Table 3; Fig. 5).

**Table 3:** Showing Post hoc pairwise comparison (P-value between every two groups)

	Group II (experimental) after 14 days	Group II (experimental) after 25 days
Group I (control group)	< 0.001	< 0.001
Group II (experimental) after 14 days		< 0.001

## Discussion

Bronchial asthma is a heterogeneous respiratory condition primarily characterized by chronic inflammation of the airways, which leads to clinical manifestations such as increased bronchial hyperreactivity, excessive mucus secretion, wheezing, coughing, chest tightness, and dyspnea. The severity and frequency of these symptoms can fluctuate over time and vary in intensity, affecting individuals across all genders, age groups, and ethnic backgrounds. In recent years, the global prevalence of asthma has risen significantly, with a notable burden of morbidity in pediatric populations and increased mortality in adults, currently affecting over 300 million individuals worldwide [31].

Meanwhile, the selection of anti-asthmatic medications is determined based on the severity of the disease and the specific type of asthma present. Pharmacological treatment primarily targets two main classes of drugs: bronchodilators and anti-inflammatory agents. Patients may be prescribed either short-term therapy to manage acute exacerbations or long-term treatment for maintenance and symptom control. Multiple studies have demonstrated that combining salmeterol and fluticasone propionate is more effective in improving lung function and controlling asthma symptoms [32, 33].

However, the majority of these therapeutic agents are administered through inhalers or nebulizers, which inevitably leads to contact with both hard and soft dental tissues, thereby elevating the potential for adverse effects [31, 34, 35]. Consequently, the current study aimed to examine the impact of the Salmeterol/Fluticasone Propionate combination therapy on the parotid glands in a rat model.

In this study, male rats were selected over females to minimize the influence of hormonal fluctuations associated with female rats’ reproductive cycles, which could potentially confound the study’s results [36]. Additionally, the αSMA antibody was employed as an immunohistochemical marker for the parotid gland. αSMA is a protein specifically expressed in myoepithelial cells and serves as a useful indicator of early cellular differentiation [37].

In the current study, examination of histological sections of group I revealed serous acini with a nearly spherical shape and basally situated nuclei. Striated duct lining showed short columnar cells with centrally placed nuclei, regular lining thickness, and a clear lumen. This result agrees with [38] description of a rodent’s normal histological structure of the parotid gland.

The histological results of Group II after 14 days showed serous acini nearly spherical in shape with basally situated nuclei. Some acini showed spacing with hyperchromatic nuclei. Striated ducts showed an apparent thinning of the duct lining and mild stagnant secretion. In contrast, excretory ducts showed nearly regular pseudostratification at some parts, apparent epithelium thinning at other parts, hyperchromatic nuclei, and almost mild cytoplasmic vacuolations. These results come in parallel with the histological findings of [39], who studied the effect of selective β2 agonists on rats’ parotid glands after 7 and 15 days of treatment.

The authors reported dense areas of chromatin at the periphery of nuclei and cytoplasmic vacuolations. They attributed these findings to the destructive effect of β2 agonists on parotid acinar cells, causing cellular changes similar to those preceding cellular apoptosis. These results agreed with [40], who reported that treating human embryonic kidney 293 cell lines expressing exogenous ß2 adrenergic receptors with β2 agonists induces early cellular apoptosis. Moreover [41], reported that the presence of cytoplasmic vacuolations may be explained by the effect of β2 agonists on the cellular mechanisms that regulate exocytosis, causing the retention of cytoplasmic vacuoles in acinar cells.

Furthermore, our findings were aligned with a study by Taher et al. [42], which demonstrated significant acini shrinkage, distortion in the arrangement of acinar cells of submandibular glands, pyknosis of the nuclei, and the presence of variable-size vacuoles in most acinar cells, and changes occurred in the striated ducts after rats were administered corticosteroids for 2 weeks. Also, they explained the presence of vacuoles in the cytoplasm of the acinar cells due to local cellular degeneration, where corticosteroids act mainly on the metabolism of gland cells.

Moreover, in the current study, Group II, after 25 days, histological results showed serous acini with relatively irregular outlines and hyperchromatic nuclei. Some spacing between serous acini was seen. Excretory ducts showed an apparent thinning with an obvious inflammatory cell infiltration and multiple extravasated blood vessels. These results came in agreement with Metwally et al. and Ochiai et al. [23, 43], who reported similar cellular changes in the duct system of rats’ submandibular and parotid glands after 25 days of treatment and even after the cessation of treatment with β-adrenergic agonists.

The authors attributed these results to the long-term treatment with β-adrenergic agonists, which induce destructive morphological changes and apoptosis in the acinar and ductal cells of rats. These results came in parallel with [39], who stated that prolonged use of β2 agonists might destroy cellular rough endoplasmic reticulum (RER), inducing a process named RER stress. Prolonged RER stress triggers the start of a cell-death program, which may explain the histological features of acinar destruction of group II after 25 days in the current study.

Moreover, the study conducted by Sag et al. [44] demonstrated that following one month of combination therapy with a long-acting β2-agonist and a corticosteroid in pediatric and adolescent individuals with asthma, the mean salivary secretion rate significantly reduced.

Meanwhile, according to Barnes et al. [45], prolonged use of β2 agonists causes down-regulation of β2 receptors. However, the combination of corticosteroids and β2 agonists enhances the expression of β2-receptors and protects them from down-regulation due to prolonged exposure to β2-agonists. The author attributed this effect to the potential desensitization of β2-receptors, mediated by the release of the enzyme G-protein receptor kinase-2 (GRK-2), which uncouples the receptor, leading to the loss of β2-agonist efficacy. Nevertheless, this effect can be reversed by the action of corticosteroids.

Although the synergistic combination of corticosteroids and β2 agonists may improve asthma treatment by enhancing the effect of β2-agonists, this leads to an overall increase in intracellular cAMP levels, which has been shown to reduce the presence of CD8 + cells and macrophages in individuals with COPD [46]. Mohiti et al. [47] found that corticosteroids lower salivary pH and increase viscosity, leading to quantitative and qualitative alterations in saliva. These changes can negatively impact oral and dental health; therefore, greater attention should be given to the oral health of individuals using corticosteroids.

In the current study, all groups had a statistically significant difference. In group I, the immunohistochemical and statistical results showed a positive immunoreaction with a strong intensity of myoepithelial cells at the periphery of serous acini. This finding agrees with Alali and Kochaji [48],who reported that the proliferation of myoepithelial cells in normal parotid glands preserves the physiological regeneration of the acini and intercalated ducts.

In the current study, the immunohistochemical and statistical findings of Group II after 14 days represented myoepithelial cells with a positive immunoreaction of moderate intensity around serous acini, while Group II, after 25 days, showed a positive immunoreaction of mild intensity. These results came in parallel with the immunohistochemical results of Ochiai et al. [42], who reported a marked decrease in the BrdU immunohistochemical staining of rats’ acinar cells of submandibular glands after a 10-day administration of a non-selective β-adrenergic receptor. In addition, this could be attributed to the cessation of DNA synthesis in parotid acinar cells after the 11th day of treatment with β2 agonists [23].

Moreover, our results agreed with those of Taha et al. [49], whose immunohistochemical findings demonstrated strong caspase and weak PCNA immunoreactivity in the rats’ submandibular salivary glands after they were treated with dexamethasone (corticosteroids) for 30 days. That is due to corticosteroids inducing apoptosis and reducing the degree of cellular proliferation and DNA synthesis in the cell cycle.

### Limitations

This study has certain limitations, including first, no separate groups receiving salmeterol or fluticasone, which would have allowed more precise differentiation of the individual effects of each drug. Second, the intraperitoneal route of administration, although selected to ensure accurate control of the administered dose and achieve consistent systemic exposure in all experimental animals, does not mimic the clinical inhalation pathway. In addition, the quantification of α-SMA was primarily based on area percentage and complemented by qualitative intensity assessment; future studies employing advanced morphometric techniques could provide a more comprehensive evaluation.

## Conclusions

To our knowledge, the findings of this study provide the first histological and immunohistochemical evidence of parotid gland alterations, suggesting a potential impact on salivary function resulting from the prolonged use of a salmeterol/fluticasone combination therapy. By revealing structural and functional changes, this study expands current understanding of the systemic implications of asthma treatment. It underlines the importance of interdisciplinary care for patients on long-term inhaled medications. Regular follow-up of oral health in these individuals should be considered. Based on the obtained results, the present study concluded that long-term treatment with a selective β2 agonist (salmeterol), along with corticosteroids (fluticasone propionate), attempting to control asthmatic symptoms, still induces destructive effects in parotid acinar and ductal cells. 

## Data Availability

All data generated or analyzed during this study are included in this published article.

## Abbreviations

COPD: Chronic obstructive pulmonary disease
ICS: Inhaled corticosteroids
GINA: Global Initiative for asthma
Sal: Salmeterol
IL-4: Interleukin-4
MASRI: Medical and scientific research institute
α-SMA: Alfa smooth muscle actin
